# Looking Back

**Published:** 1904-01-02

**Authors:** 


### The Hospital.

A Journal of

The Medical Sciences and Hospital Administration.

Vol. XXXV.—No. 901. JANUARY 2, 1904.

### Looking Back.

Before this page falls under the eyes of those who will be its readers, "the old sexton, Time," will already have claimed the third year of the twentieth century as his property, and will be engaged in consigning it to the grave of its predecessors. We may follow it, if not exactly as mourners, yet in all seriousness, and may ask ourselves how far the medical profession, and we ourselves as medical journalists, and the great charities which, by virtue of our title, we may in some measure assume to represent, have shared in the progress, or have derived benefit from the lessons which a year, however uneventful, can scarcely fail either to bring or to teach. The retrospect presents nothing startling; but, on the whole, it is one which may be regarded with satisfaction. The sculptor, who was told that his work had made no progress, pointed out that he had modified this curve, that he had softened that outline, that he had given a fuller meaning to such or such an expression; and, when it was objected that these things were trifles, replied that they were contributory to perfection, and that perfection was not a trifle. Perhaps the chief feature of the year, as far as medical science is concerned, has been an increased general recognition of the necessity of methodising research, as the principal means by which we may hope to find out the secrets of nature; and this increased recognition has been largely brought about by the proceedings and the reports of the Cancer Fund. These proceedings and reports, as far as the public are concerned, have been educational in a high degree; showing the absolute futility of hasty conclusions and the tendency to error inseparable from limited experience, as well as the fact that the well of truth can only be sounded or explored by agencies commensurate with its profundity. The operations of the Fund have already shown that, in the language of the Hebrew prophet, we must cease to do evil before we can learn to do well, and that it is necessary to abandon the pursuit of *ignes fatui* as a preliminary to any endeavour to follow the guiding light of fact. In the particular case in question it has been found possible to throw overboard a whole series of hastily-formed hypotheses, and, at least for the present, to admit that no extrinsic cause of cancer can reasonably be looked for. There seems to be no invader from without, and hence no frontier that can be defended.

When we turn from the absolute growth of medical science to the arrangements by which it is rendered available for the wants of the great mass of the community there can be no doubt that the year has brought about an increasing willingness to consider and treat the hospital system as a whole, and so to abolish the rivalry, or almost antagonism, which has too long existed between its component parts. The amount of the demands certain to be made upon Londoners for the support of their hospitals during the coming year, has lately been estimated by the Prince of Wales at more than a million sterling; and even the greater part of this estimate is for extraordinary expenses, such as those incidental to reconstruction or to removal, and leaves out of account the daily cost of maintenance. In such circumstances the time has evidently come for some endeavour to equalize and distribute the burden, and for the abolition of all methods of collection that are based upon or contributory to the rivalry between institutions or between localities. The *Times* paper, which for many years has fully recognised the value and importance of hospitals as national institutions, and has given great prominence to their claims and uses, published last week a letter from an obviously well-informed correspondent, urging the necessity for the formation, in London, of some organisation for central hospital control, and suggesting that at least the commencement of such an organisation might be established by the executive bodies of the great collecting funds, which, even now, are guided in their distribution by considerations of the cost and the usefulness of the various institutions upon their lists. Unless something of this kind can be accomplished, the correspondent in question maintains that a financial breakdown is inevitable.

The correspondent of the *Times* is not justified in his pessimism, as we show in an article on another page. No such financial breakdown is imminent or remotely probable, as the figures prove.

We are glad for the sake of the public and of the hospitals that this should be so; for municipal subvention, or, in other words, casting the hospitals upon the rates, and so bringing them under the control of the Councils of the boroughs in which they are respectively situate, would be an evil in many ways. The result of such a change would be, of course, that a mere subvention would soon cease to be sufficient, and that the voluntary contributor would cease to exist. He would say that, if the rates were to be called upon for part of the expenditure, they ought in common justice to be called upon for the whole, so that every inhabitant might contribute, according to his presumed means, to establishments in the benefits of which all might claim a right to share. Physicians and surgeons would then be selected with reference to local politics, with reference to their views as to the best course for a projected tramway, or in dependence upon their power of rendering themselves professionally acceptable to the wives of aldermen. Preliminary medical teaching is fast being diverted from the metropolis to provincial centres; and, in the case supposed, the clinical teaching would soon follow the preliminary, and the great medical school of London would cease to exist. The only way to avoid the occurrence of such evils is by economy of expenditure; and the only way of enforcing economy, as far as we can see, is by such an amount of co-operation as may at least furnish a safeguard against waste.

